# DLAAD-deep learning algorithms assisted diagnosis of chest disease using radiographic medical images

**DOI:** 10.3389/fmed.2024.1511389

**Published:** 2025-03-07

**Authors:** Mosleh Hmoud Al-Adhaileh, Bayan M. Alsharbi, Theyazn H. H. Aldhyani, Sultan Ahmad, Mohammed Amin Almaiah, Zeyad A. T. Ahmed, Saad M. AbdelRahman, Elham Alzain, Shilpi Singh

**Affiliations:** ^1^Deanship of E-Learning and Distance Education and Information Technology, King Faisal University, Al-Ahsa, Saudi Arabia; ^2^Department of Information Technology, College of Computers and Information Technology, Taif University, Taif, Saudi Arabia; ^3^Applied College, King Faisal University, Al-Ahsa, Saudi Arabia; ^4^Department of Computer Science, College of Computer Engineering and Sciences, Prince Sattam Bin Abdulaziz University, Al-Kharj, Saudi Arabia; ^5^School of Computer Science and Engineering, Lovely Professional University, Phagwara, India; ^6^King Abdullah the II IT School, The University of Jordan, Amman, Jordan; ^7^Department of Computer Science, Dr. Babasaheb Ambedkar Marathwada University, Aurangabad, India; ^8^Amity School of Engineering and Technology, Amity University, Patna, India

**Keywords:** deep learning, medical images, diagnosis, chest diseases, radiography

## Abstract

**Introduction:**

Viral infections can cause pneumonia, which is difficult to diagnose using chest X-rays due to its similarities with other respiratory conditions. Current pneumonia diagnosis techniques have limited accuracy. Novelty, of this research is developed a application of deep learning algorithms is essential in enhancing the medical infrastructure used in the diagnosis of chest diseases via the integration of modern technologies into medical devices.

**Methods:**

This study presents a transfer learning approach, using MobileNetV2, VGG-16, and ResNet50V2 to categorize chest disorders via X-ray images, with the objective of improving the efficiency and accuracy of computer-aided diagnostic systems (CADs). This research project examines the suggested transfer learning methodology using a dataset of 5,863 chest X-ray images classified into two categories: pneumonia and normal. The dataset was restructured to 224 × 224 pixels, and augmentation techniques were used during the training of deep learning models to mitigate overfitting in the proposed system. The classification head was subjected to regularization to improve performance. Many performance criteria are typically used to evaluate the effectiveness of the suggested strategies. The performance of MobileNetV2, given its regularized classification head, exceeds that of the previous models.

**Results:**

The suggested system identifies images as members of the two categories (pneumonia and normal) with 92% accuracy. The suggested technique exhibits superior accuracy as compared to currently available ones regarding the diagnosis the chest diseases.

**Discussion:**

This system can help enhance the domain of medical imaging and establish a basis for future progress in deep-learning-based diagnostic systems for pulmonary disorders.

## 1 Introduction

Pneumonia is a lung inflammation that causes cough, fever, and breathing difficulties. Prompt diagnosis is crucial to successful treatment and improved prognoses. Pneumonia is a pulmonary condition, so radiographic findings may not consistently validate a diagnosis of pneumonia. Consequently, even with the use of contemporary technology, it remains challenging to definitively differentiate pneumonia from other pulmonary conditions based solely on radiographic criteria (1).

Viral infections, including pneumonia, have long threatened human health, with both viral and bacterial infections harming lung function. Pneumonia commonly presents with symptoms such as discomfort, cough, and dyspnea. Pneumonia affects around 7% of the global population annually, making timely diagnosis essential, as with most medical conditions. As a result, efforts to categorize medical images automatically have significantly increased (2). This research project focuses on classifying medical images, with deep learning (DL) emerging as a leading method for such tasks (3). Furthermore, DL models have exhibited enhanced performance relative to traditional methods in the analysis of chest diseases to detect pneumonia (4, 5).

The DL architectures used have displayed enhanced analytical performance, exceeding that of medical professionals (6). Chest X-ray images have been employed for various applications utilizing DL models, including the identification of tuberculosis (7), the segmentation of tuberculosis (8), large-scale recognition (9), the detection of COVID-19 (10, 11), and the categorization of radiographs (12). Diagnosing diseases by using chest images in DL models, and it is progressing swiftly, and appropriate regions of interest can be identified in these images, allowing the models to detect pneumonia (13). Additionally, the application of DL models addresses problems that typically require significant time to resolve using traditional methods. However, these DL models require many precisely labeled training data. Pneumonia has a high prevalence in developing nations, which are marked by overcrowding, pollution, and unsanitary environmental conditions. This high prevalence has led to a lack of medical supplies in those nations. As a result, timely identification and intervention could help prevent this illness from advancing to a terminal condition.

The diagnostic evaluation of the lungs with radiographic techniques often involves various medical images, such as X-ray radiography. X-ray radiography is a crucial and often economical diagnostic technique used for evaluating the lungs. The areas that appear white in the pneumonic area on images generated by medical devices are referred to as infiltrates. Chest imaging diagnostics for pneumonia detection are subject to variability based on interpretation. Thus, an automated detection method is essential.

The DL approach represents a robust artificial intelligence methodology that is capable of efficiently addressing intricate computer vision challenges. Convolutional neural networks (CNNs) are often utilized for image categorization applications. Data-intensive models require a substantial volume of data to achieve maximum performance. This need poses a challenge for biological image classification because it demands the engagement of proficient clinicians to annotate each image. Transfer learning is a strategy that can be used to tackle this issue (14, 15). This method uses a model trained on an extensive dataset to address the problems related to using a smaller dataset, using the network weights derived from the original model (16, 17). In this research project, we use several pre-trained networks to identify early-stage pneumonia, and we then outline the subsequent contributions.

- The chest X-ray examination is the most prevalent medical imaging test globally, and it is essential in identifying common thoracic disorders.
- X-ray interpretation is a labor-intensive process, and there are too few well-educated radiologists in several healthcare systems.
- Deep learning algorithms designed for the diagnostic interpretation of chest diseases have not been evaluated against the performance of competent human radiologists.

### 1.1 What are findings of this research?

- We developed a DL technique that concurrently identifies two clinically relevant disorders using chest X-rays.
- We evaluated the approach with a validation set of 5,863 X-ray images.
- The MobileNetV2 model has 92% accuracy in terms of identifying thoracic ailments.
- Using a single institutional dataset, DL systems can identify certain anomalies in chest X-rays with an accuracy comparable to that of professional radiologists.
- The validation of DL algorithms, such as the one presented in this paper, may allow for the immediate, high-quality analysis of chest radiographs.

## 2 Related work

Over the last decade, numerous developers and scientists have employed ML and DL methodologies to systematically detect lung infections through the analysis of chest diseases. CheXNet is a sophisticated 122-layer CNN framework developed and diagnosis by Sirazitdinov et al. (18). This methodology was formulated based on an extensive dataset of chest images representing 14 medical conditions. These medical chest images were analyzed with ChexNet, and the results were compared with another models. The DL-based CNN method exceeded the average effectiveness regarding the identification of radiographic pneumonia. A CNN approach was developed to select features from medical chest images, resulting in improved classifier performance regarding pneumonia detection as compared to previous methods that relied on manual feature extraction. Hussain et al. (19) introduced a method combining adaptive average-filtering CNNs with random forests for pneumonia diagnosis. This adaptive filtering helps reduce image noise, enhancing classification accuracy. The CNN model, which consists of two layers and uses dropout techniques, also benefits, in this regard, from further preprocessing with adaptive filtering. Despite their advantages, CNN models do require a significant number of labeled data to obtain optimal training outcomes.

Wang et al. (20) utilized a COVID dataset related to novel coronavirus pneumonia and presented the COVID-Net model, which incorporates variability in network architecture and has shown better performance than VGG19 and ResNet50. Shaban et al. (21) introduced a hybrid diagnostic method that prioritizes key attributes by mapping them within the patient space, creating a feature connectivity graph (FCG) to highlight each feature’s importance and connections with other features. Ozturk et al. (22) developed DarkCovidNet, a model designed to automatically detect COVID-19 using raw chest X-ray images, allowing the accurate binary classification of chest disease patients and non-infected individuals, as well as the trinary classification of COVID-19 patients, pneumonia patients, and non-infected individuals. The model exhibited improved performance by leveraging DarkNet-19 as a base framework. Analyzing the differences between the original DarkNet-19 and DarkCovidNet, it is evident that DarkCovidNet utilizes a reduced number of layers and filters, which leads to a notable improvement in performance. Li et al. (23) developed an innovative voting system that effectively classifies images into four chest diseases. They used CNNs to develop an AI model focused on improving data adaptability, ultimately employing a majority rule decision-making strategy for the diagnosis of chest diseases. Bhandari et al. (24) introduced a streamlined CNN approach designed for the classification of various types of chest diseases, namely pneumonia chest dieses, tuberculosis pneumonia chest dieses, and normal conditions. A unique DL system, Pneumonia-Plus, has been developed to accurately diagnose various types of pneumonia by analyzing CT data (25). The study presented in Yi et al. (26) offers a CNN approach that is scalable, interpretable, and aimed at automating pneumonia detection through the analysis of chest images. The proposed CNN model demonstrates superior capabilities in terms of feature extraction and accurately classifies images into two categories (normal and pneumonia), outperforming existing methods based on extensive evaluations across multiple performance metrics. The research presented in Goyal and Singh (27) was instrumental in identifying complex lung diseases.

Wang et al. (28) utilized a chest dataset comprising eight disease diagnoses and multiple annotations for each image. Several natural-language-processing techniques are employed to identify problematic phrases, remove negation, and minimize ambiguity. The experiments employ CNNs to distinguish between eight thoracic diseases. Guan et al. (29) utilized a DL model called the attention-guided (AG-CNN) to differentiate between eight types of cancer identified in prior studies. Singh et al. (30) evaluated the effectiveness of DL methods in detecting abnormalities in standard frontal chest radiographs and monitoring the stability of the results over time. Later studies applied a methodology utilizing CNNs for the detection of pneumonia through chest radiography (31, 32).

## 3 Methodology

**Figure 1:**
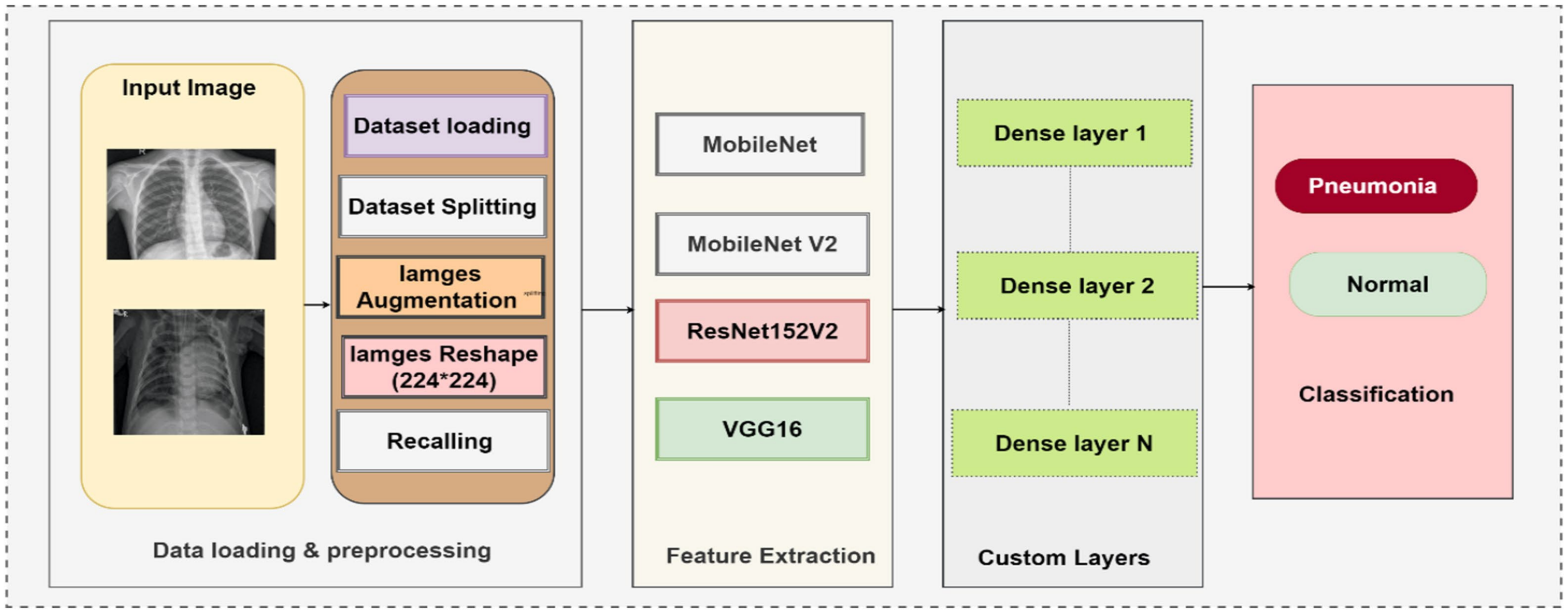
Framework of diagnosis chest.

This study presents the development of an innovative DL approach to chest disease detection utilizing MobileNet, VGG16, MobileNetV2, and ResNet50V2. This model underwent training and evaluation utilizing images representing two significant chest conditions, namely pneumonia and “normal” class. We utilized image data generators to train a DL model with chest X-ray images, resizing them to 224 × 224 pixels. The images in the dataset underwent normalization as a pre-processing step, followed by the essential modification of the data using the categorical variables for the proposed DL models. The dataset pertaining to chest diseases was systematically divided into three distinct categories: testing, training, and validation. Figure 1 depicts the chest diseases proposed framework.

### 3.1 Dataset

**Figure 2:**
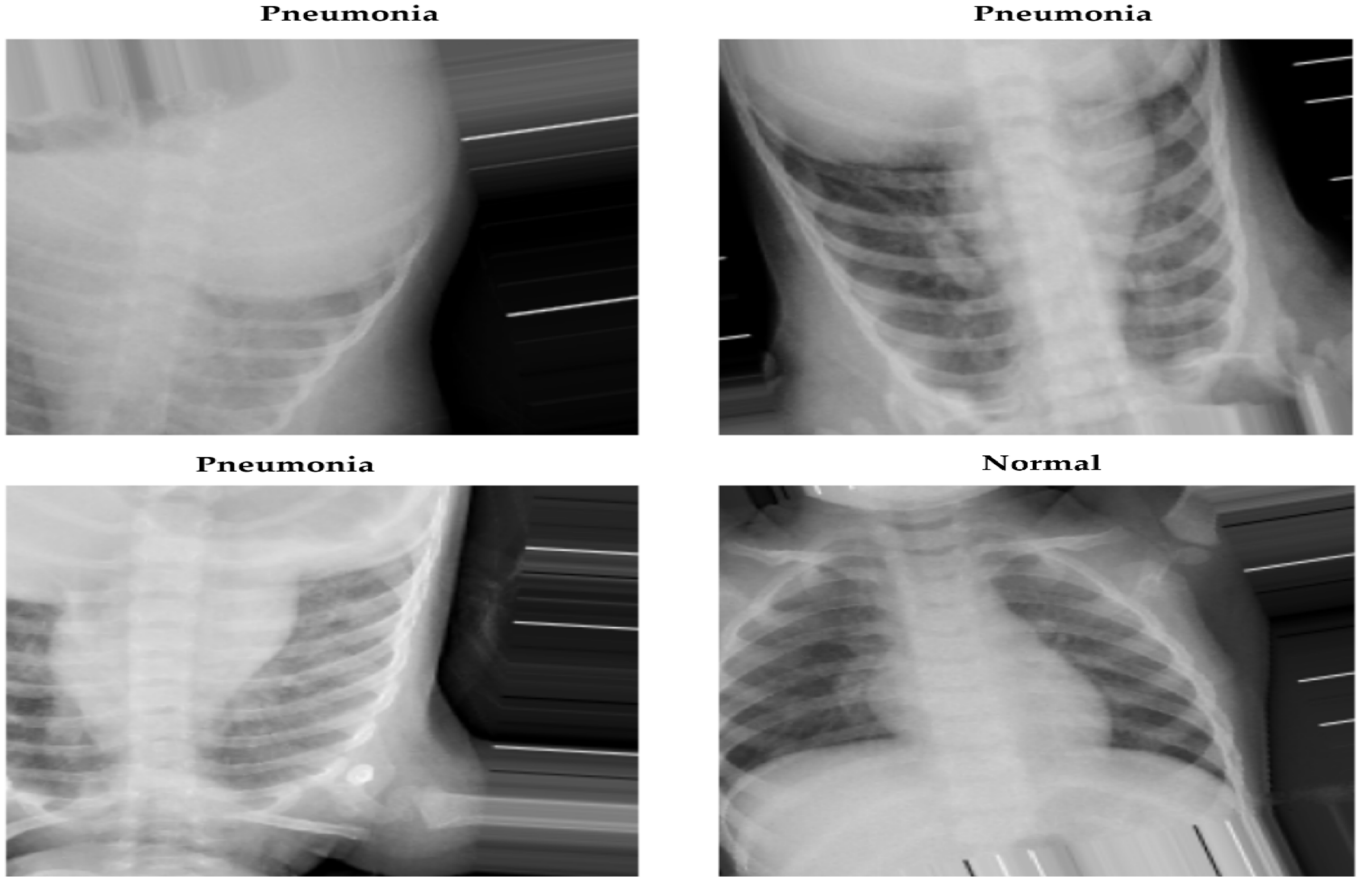
Chest X-ray images.

The datasets were acquired from the public repository Kaggle. The collection includes 5,863 JPEG X-ray images classified into two categories: pneumonia and normal. Anterior-posterior chest X-rays were sourced from retrospective cohorts of pediatric patients aged 1 to 5 years who received treatment at the Guangzhou Women and Children’s Medical Center. These images were taken as part of the standard medical procedure for patients. Figure 2 presents a sample of these medical images.

### 3.2 Preprocessing

To improve model generalization and prevent overfitting, data augmentation is applied in the DL model used to classify the chest X-ray images. The image dimensions are configured to 224 × 224 pixels, which are appropriate for models that require fixed input sizes. The training data are enhanced by using the parameters of augmentation methods, such as rescaling pixel values, rotating images by 20 degrees, altering image width and height by 25%, zooming, and flipping the images both horizontally and vertically. These changes enhance the model’s generalizability by having the images mimic real-world variance.

### 3.3 Normalization

The pixel values were rescaled by a factor of 1/255 using the standardized normalization approach to restructure the dataset. This normalization process transforms the pixel intensity values from a range of 0 to 255 to a range of 0 to 1. Normalizing the data in this manner enhances the model’s learning efficacy by mitigating the problems associated with excessive variation in the input data.

### 3.4 Splitting data

**Figure 3:**
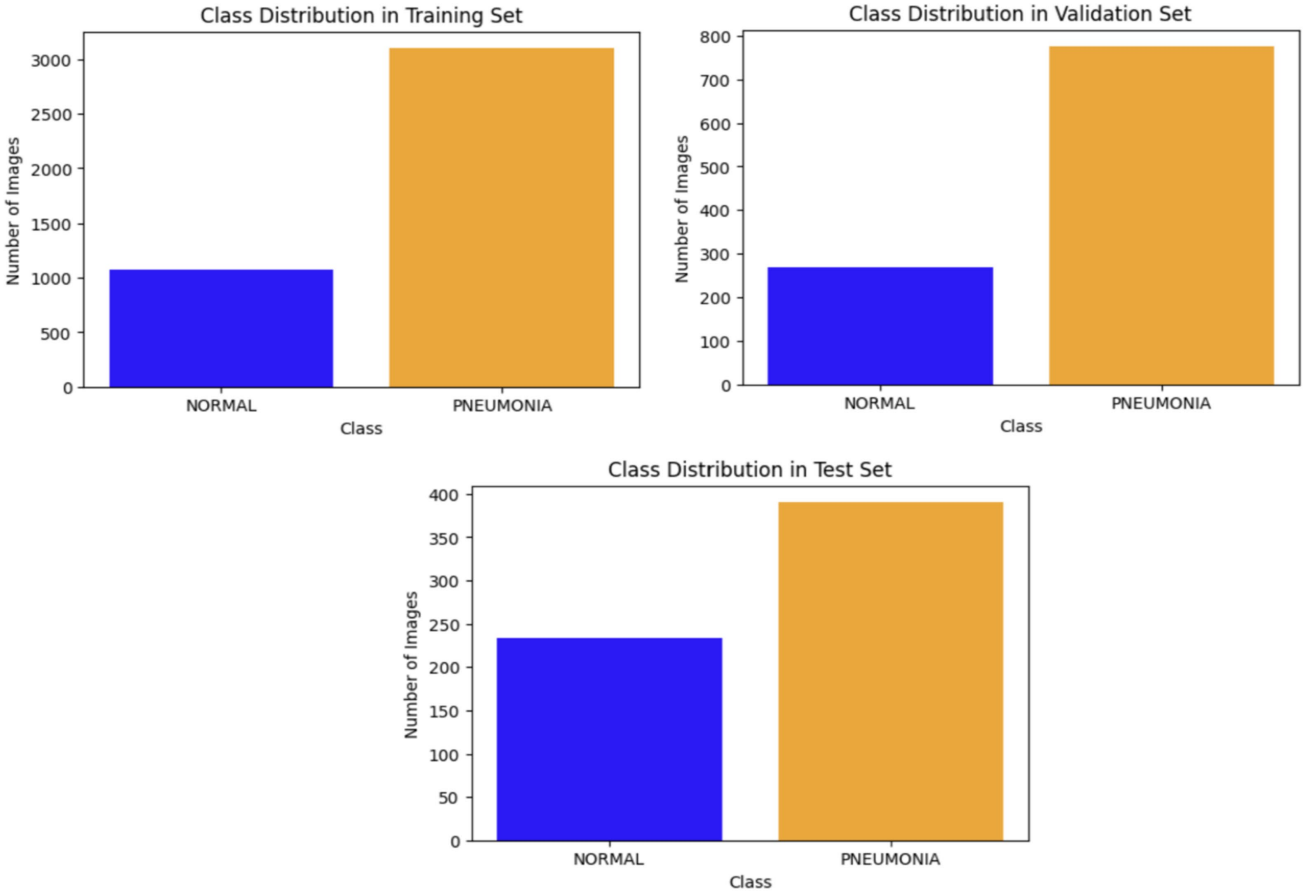
Classification of X-ray images.

Data are generally classified into three primary subsets: training, validation, and testing. The training phase is crucial in effectively fitting DL models, with 80% of the total data being designated for this purpose. The validation set serves to tune the model during training by assessing its performance on data not encountered during the training phase, thereby safeguarding the integrity of the model’s learning. Meanwhile, 20% of the collection is set aside for testing, allowing for the evaluation of the model’s final performance on new real-world data. This test set is separate from both the training and validation processes. Figure 3 shows how the X-ray images obtained from the dataset are classified.

### 3.5 Deep learning models

#### 3.5.1 MobileNetV2 model

In its architecture, MobileNetV2 was proposed for diagnosing the medical images. Because the input and output of the leftover blocks are narrow bottleneck layers, this structure is defined by this characteristic. Additionally, MobileNetV2 makes use of lightweight convolutions to filter features in the expansion layer. This is done from the perspective of the expansion layer itself. The elimination of non-linearities in the thin layers is the final benefit of this process and certainly not the least significant. MobileNetV2 is a pre-trained DL architecture that is used as a feature extractor for picture categorization tasks. The MobileNetV2 model, which is pretrained on ImageNet, is imported without its classification layer, and its weights are fixed to inhibit further training. The extractor features are processed by MobileNetV2 and then sent via a global average pooling layer, which reduces the dimensionality of the feature maps while preserving essential information. A custom classifier is constructed using thick layers for the purpose of classification. The layers are entirely interconnected via ReLU activations and decrease in size to 512, 256, and 128 units. Each dense layer is succeeded by batch normalization to enhance stability and accelerate the training process, along with dropout layers, utilizing dropout rates of 0.5, 0.3, and 0.2, to mitigate overfitting. Early halting and model checkpointing are used to prevent overfitting and preserve the optimal model. The Adam optimizer was employed to construct the model. The significant indicators utilized to enhance the MobileNetV2 model’s ability to classify chest images according to the diseases present are shown in Table 1 (see Figure 4).

**Table 1:** MobileNetV2 parameters.

# Features	Values
Reshape of image	224, 224, 3
Complied function	Adam
Epochs	20
Dropout_rate	0.5, 0.3, 0.2
Dense_layers	512, 256, 128
Function	ReLU

**Figure 4:**
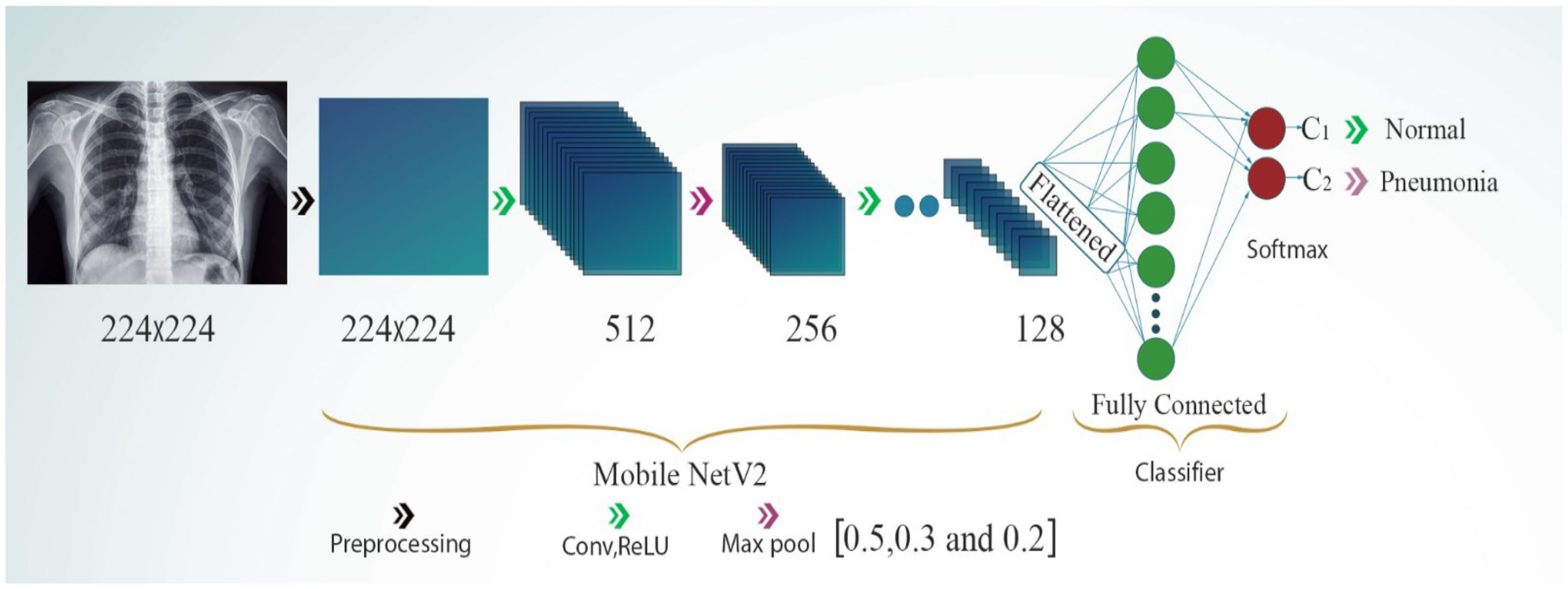
MobileNetV2 model.

#### 3.5.2 The VGG-16 model

**Figure 5:**
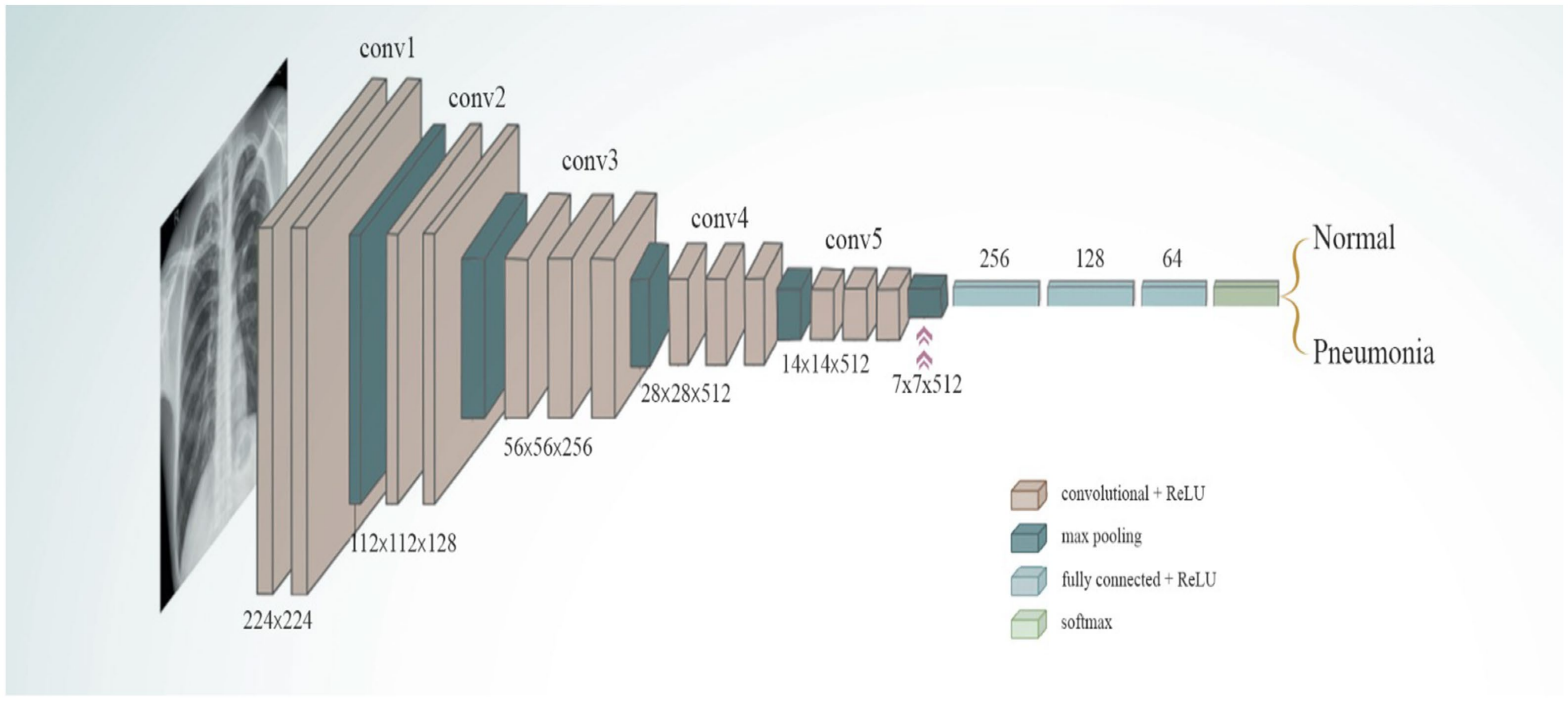
VGG-16 mode.

The VGG-16 model is a DL architecture. It was developed by the Visual Geometry Group (VGG) at Oxford University. It has 16 layers in total, comprising 13 convolutional layers and three fully connected (dense) layers. The VGG-16 model is known for its simplicity and efficacy, as well as its capacity to attain robust performance across healthcare systems, including medical image categorization and object identification in medical images. The VGG-16 architecture is presented in Figure 5. To accomplish binary classification tasks, this model employs the pre-trained VGG-16 architecture as the foundation for the extraction of image features. This architecture serves as the backbone of the algorithm. To pre-train the VGG-16 model, ImageNet is used, and the model is loaded without its top layer, which is the classification layer.

When constructing the VGG-16, a custom classifier, the building procedure included the use of three thick layers that were activated using ReLU. The first dense layer has 256 neurons, while the second dense layer has 128 neurons, and the third dense layer has 64 neurons. The first dense layer is the densest. To prevent the model from becoming too accurate, it was designed with dropout layers, which had dropout rates that varied between 0.45 and 0.3 units. The purpose of the final output layer is binary classification. This final output layer consists of a single neuron that is stimulated with a sigmoid function form and designed for this purpose. To optimize the loss function, the Adamax optimizer is applied, and binary cross-entropy is utilized to compile the model. The significant indicators utilized to enhance the VGG-16 model’s ability to classify chest diseases are presented in Table 2.

**Table 2:** VGG-16 parameters.

# Features	Values
Reshape of image	224, 224, 3
Complied function	Adamax
Epochs	20
Dropout_rate	0.45, 0.30
Dense_layers 1	256
Dense_layers 2	128
Dense_layers 3	64
Learning rate	0.001
Function	ReLU

#### 3.5.3 ResNet50V2 model

**Figure 6:**
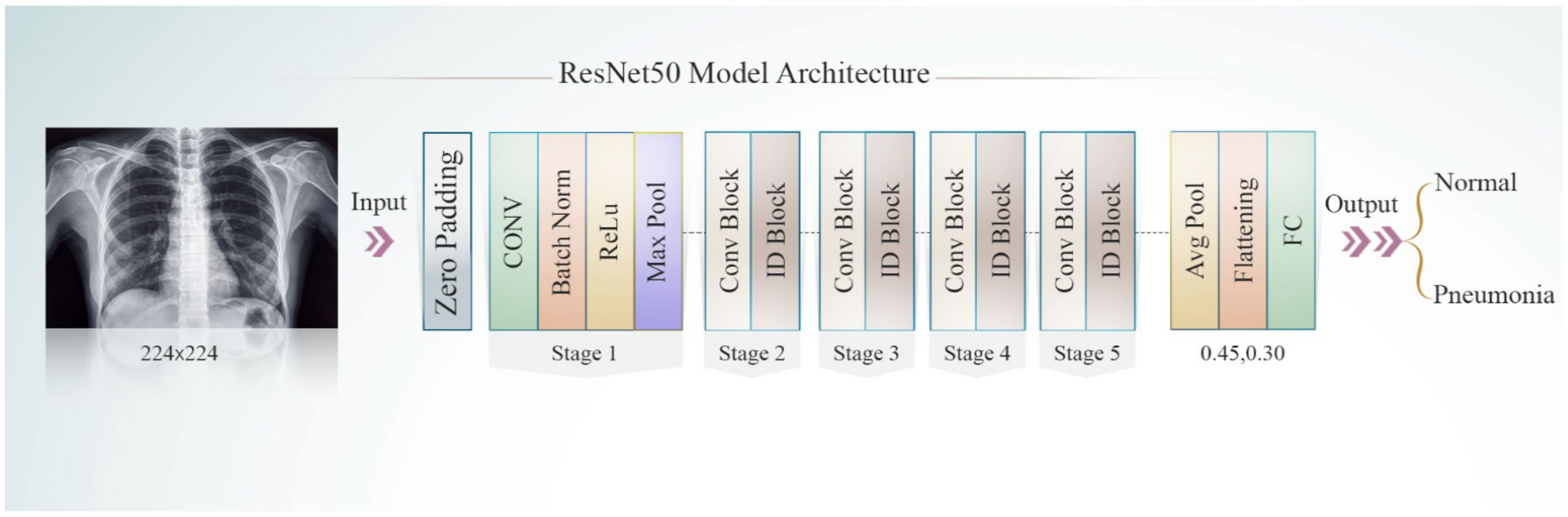
ResNet50 model.

Residual blocks are a concept devised for use in this architecture to address the problem of disappearing or expanding gradients. ResNet50V2 uses skip connections, and these skip connections allow the activation of one layer to be linked to the activation of succeeding layers passing specific intermediate layers. This can help generate a residual block. ResNet network employs a strategy that prioritizes learning over the underlying mapping, rather than having the layers learn the mapping. The development of the ResNet50V2 model is presented in Figure 6.

ResNet50V2, a powerful feature extractor, is pre-trained on the ImageNet dataset and serves as a tool for feature extraction. Throughout the training process, the pre-trained layers remain static, except for the four final layers. This inhibits modifications, using the traits that have already been acquired instead. In the ResNet50V2 model, three thick layers are incorporated: the first consists of 256 neurons, the second has 128 neurons, and the third contains 64 neurons. To mitigate model overfitting, the dropout layer is set to values between 0.3 and 0.45. The Adamax optimizer was used for model compilation. The ResNet50V2 model undergoes training on the dataset for 20 epochs. After training, the best-performing model is selected for assessment. A classification report is then produced to measure the ResNet50V2 accuracy. The significant indicators utilized to enhance the ResNet50V2 model’s ability to classify chest diseases are illustrated in Table 3.

**Table 3:** ResNet50V2 parameters.

# Features	Values
Reshape of image	224, 224, 3
Complied function	Adamax
Epochs	20
Dropout_rate	0.45, 0.30
Dense_layers 1	256
Dense_layers 2	128
Dense_layers 3	64
Learning rate	0.001
Function	ReLU

### 3.6 Evaluation metrics

In this study, four performance metrics were proposed for evaluating and testing the results of proposed architecture: accuracy, precision, sensitivity (recall), and *F*_1_-score. The mathematical formulas for these metrics are presented below Equations 1–4 display the evaluation metrics.


(1) $$\mathrm{Accuracy}=\frac{TP+TN}{FP+FN+TP+TN}\times 100$$

(2) $$F_{1}-\mathrm{score}=2*\frac{\mathrm{Precision}\times \mathrm{Recall}\;}{\mathrm{Precision}+\mathrm{Recall}}\;\times 100\%$$

(3) $$\mathrm{Recall}=\frac{\mathrm{True positives}\;}{\mathrm{True positives}+\mathrm{False positives}}\;\times 100\%$$

(4) $$\mathrm{Precision}=\frac{\mathrm{True negatives}}{\mathrm{True negatives}+\mathrm{False negatives}}\times 100\%$$

## 4 Experiment

In this research project, we constructed a system utilizing a DL model to diagnose chest diseases. We make use of medical X-ray images to evaluate the proposed method. The dataset had two classes, namely pneumonia and normal. This system achieved great accuracy and can thus assist health officials and doctors in making informed decisions about patient diagnoses.

### 4.1 Setup for the developing system

The proposed system used Python programming and was implemented using the TensorFlow library, with GPU support from the Keras library. The experiment was divided into training, testing, and validation and was conducted in the Kaggle Colaboratory environment, utilizing a Tesla T4 graphics card, 12 GB of RAM, and 66 GB of disk space. It was concluded that the model would benefit from the Adamax optimizer, a gradient descent optimization method, which is particularly effective in dealing with problems requiring extensive medical images or data.

### 4.2 Results for MobileNetV2

The results ResNet150V2 is displayed in Table 4. The ResNet150V2 model demonstrates superior efficacy in classifying chest X-ray images, effectively identifying pneumonia and differentiating normal from abnormal cases. The ResNet150V2 model attained a score of 97% according to the accuracy metric, surpassing the average accuracy. Conversely, the model achieved 82% recall and an *F*_1_-score of 89% in relation to one another.

**Table 4:** Results for the ResNet50V2 model.

Class name	Precision (%)	Recall (%)	*F*_1_-score (%)	Support/validation
Normal [0]	97	82	89	234 instances
Pneumonia [1]	90	98	94	390 instances
Accuracy			92	624 instances
Macro_Average	94	90	92	624 instances
Weighted_Average	93	92	92	624 instances

The model achieved 90% accuracy, 98% recall, and an *F*_1_-score of 94% for the pneumonia class. This signifies that the model achieved a performance that is marginally superior to the average in this category. Upon comprehensive evaluation, the model attained an accuracy of 94.90 and 92%, with the macro and weighted averages for precision, recall, and *F*_1_-score being approximately 96%. This indicates that the model performs well across both categories, making it a dependable tool for pneumonia detection in medical imaging.

**Figure 7:**
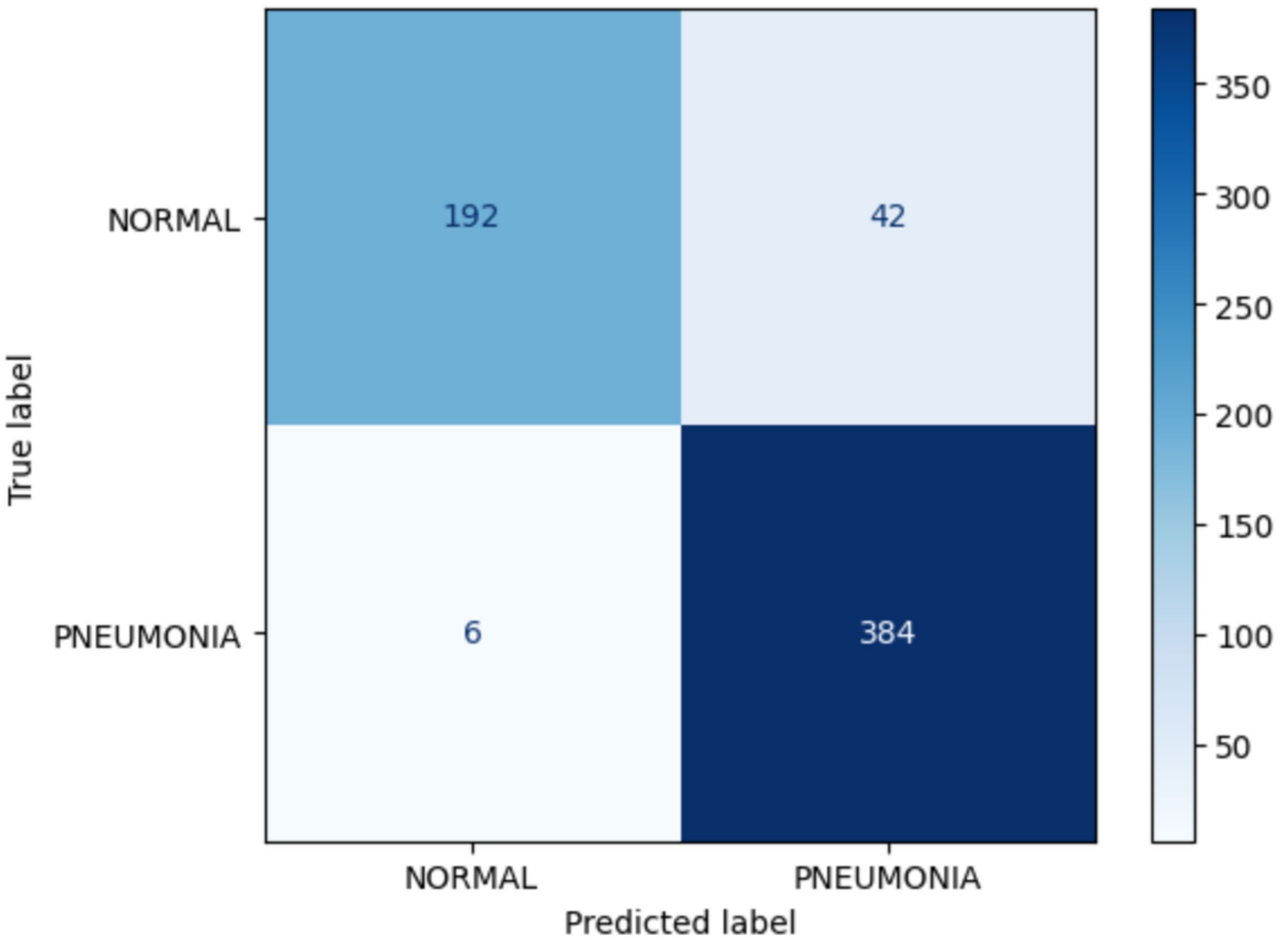
Confusion matrix for MobileNetV2.

Figure 7 confusion matrix for the MobileNetV2 model is illustrated. This matrix is used to classify the normal and pneumonia classes in the validation set of the medical chest imaging dataset. The enhanced MobileNetV2 model accurately classified 192 of 234 images as belonging to the normal class, whereas 42 images were incorrectly recognized as being indicative of pneumonia. It accurately predicted the pneumonia class in 384 of 390 images, while misclassifying six images as normal. In conclusion, we have determined that the pneumonia classification predicted by our model is superior to that for normal images.

**Figure 8:**
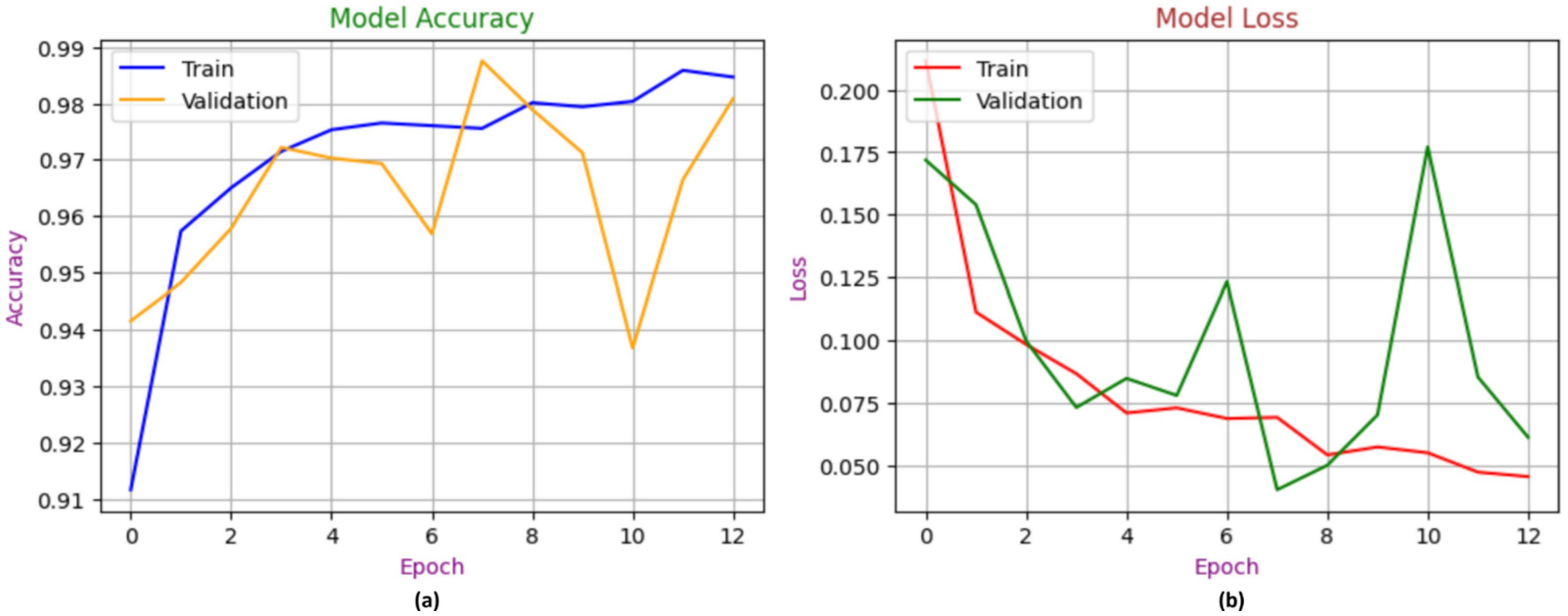
Accuracy performance of MobileNetV2, with **(A)** indicating accuracy and **(B)** indicating loss.

Figure 8 showcases the performance of the MobileNetV2 model during the process of training and validation for the identification of chest diseases using X-ray images. In contrast to the training model, which achieved 98% accuracy, the validation model began at 90% accuracy and ultimately attained 92% accuracy. The loss is a gradient that begins at 0.175 and attains a maximum value of 0.0075.

### 4.3 Results for VGG-16

The results of the computational analysis performed with the VGG-16 model are displayed in Table 5. The VGG-16 model achieved 98% accuracy in normal class, suggesting the substantial presence of true normal cases among the predicted normal cases. The VGG-16 model exhibited 72% recall, suggesting it inadequately identified a substantial proportion of true normal cases. The *F*_1_-score for the regular class was 83%. In the pneumonia category, the VGG-16 model demonstrated strong performance, achieving 85% accuracy and 99% recall, which led to an *F*_1_-score of 92%. Overall, the model achieved 89% accuracy.

**Table 5:** Results for the VGG-16 model.

Class name	Precision (%)	Recall (%)	*F*_1_-score (%)	Support/validation
Normal [0]	98	72	83	234 instances
Pneumonia [1]	85	99	92	390 instances
Accuracy			89	624 instances
Macro_Average	92	85	87	624 instances
Weighted_Average	90	89	88	624 instances

**Figure 9:**
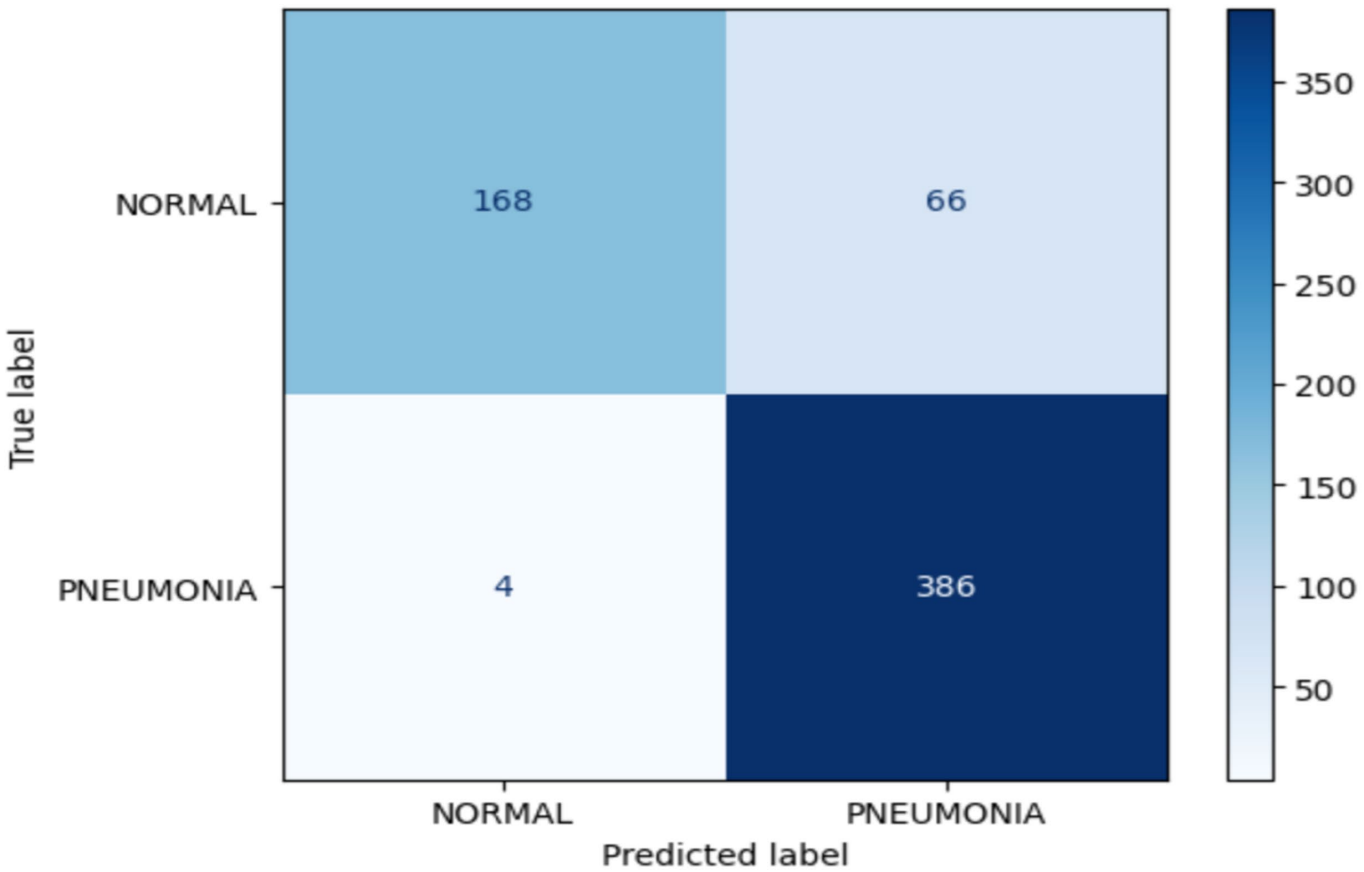
Confusion matrix for the VGG-16 model.

Figure 9 confusion matrix for the VGG-16 model is shown, highlighting its role in diagnosing chest diseases by distinguishing between normal and pneumonia classes during the validation phase. The model accurately classified 168 of 234 images in the normal category, while misclassifying 66 images as indicating pneumonia. In the pneumonia category, the model accurately identified 386 of 390 images, misclassifying four images as normal. Finally, the number of images classified as false positives (FPs) is 4 images.

**Figure 10:**
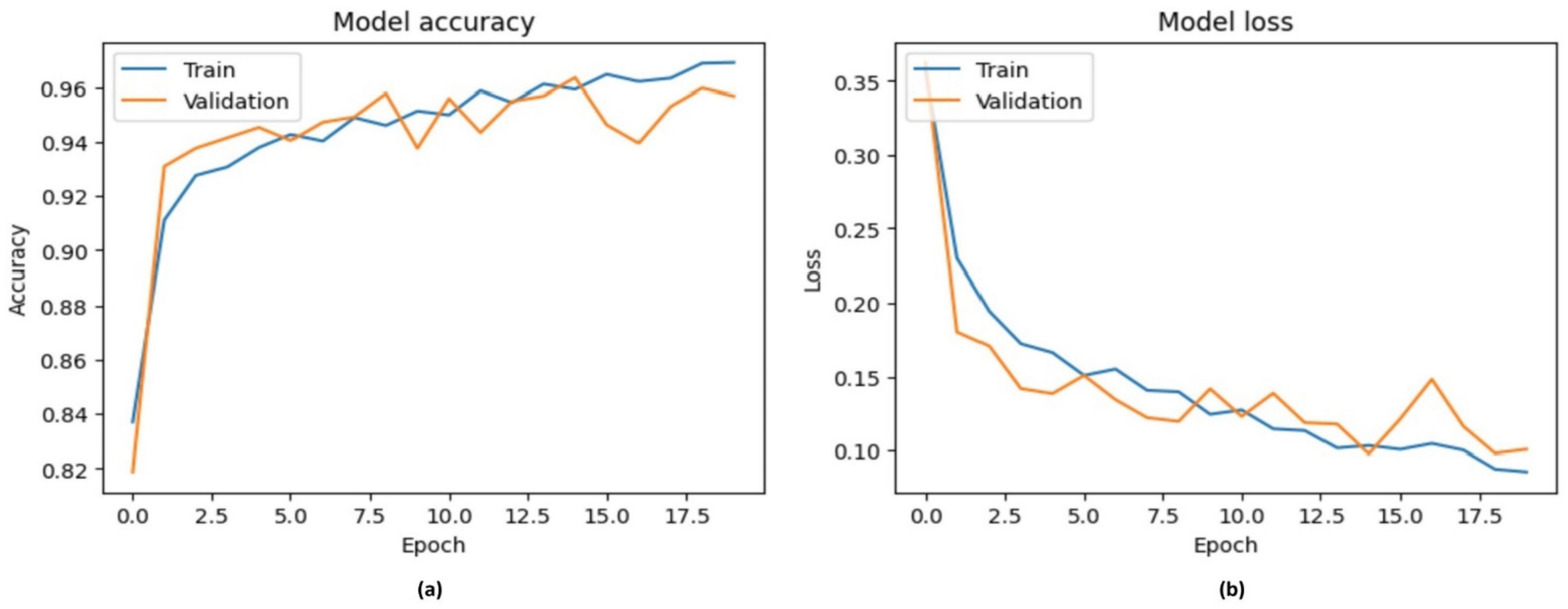
Accuracy performance of VGG-16, with **(A)** indicating accuracy and **(B)** indicating loss.

The efficacy of the VGG-16 model in identifying chest disease is seen in Figure 10. The training accuracy approaches 96%, whereas the validation accuracy stabilizes just below this figure, indicating a robust accuracy value of 89%. The loss curves show a rapid decline in both training and validation loss, ultimately stabilizing at low levels between 0.35 and 0.10, indicating good model learning and accurate predictions.

### 4.4 Results for the RestNet50v2 model

Table 6 shows the results for the ResNet50V2 model in terms of diagnosing chest diseases. This model achieved an overall accuracy of 91%. For the normal class, precision is 92%, recall is 83%, and the *F*_1_-score is 87%, indicating that the model has slightly more false negatives for this class than for the pneumonia class. For the pneumonia class, precision is 90%, recall is 96%, and the *F*_1_-score is 93%, indicating strong performance in terms of detecting pneumonia.

**Table 6:** Results for the RestNet50v2 model.

Class name	Precision (%)	Recall (%)	*F*_1_-score (%)	Support/validation
Normal [0]	92	83	87	234 instances
Pneumonia [1]	90	96	93	390 instances
Accuracy			91	624 instances
Macro_Average	91	89	90	624 instances
Weighted_Average	91	91	91	624 instances

**Figure 11:**
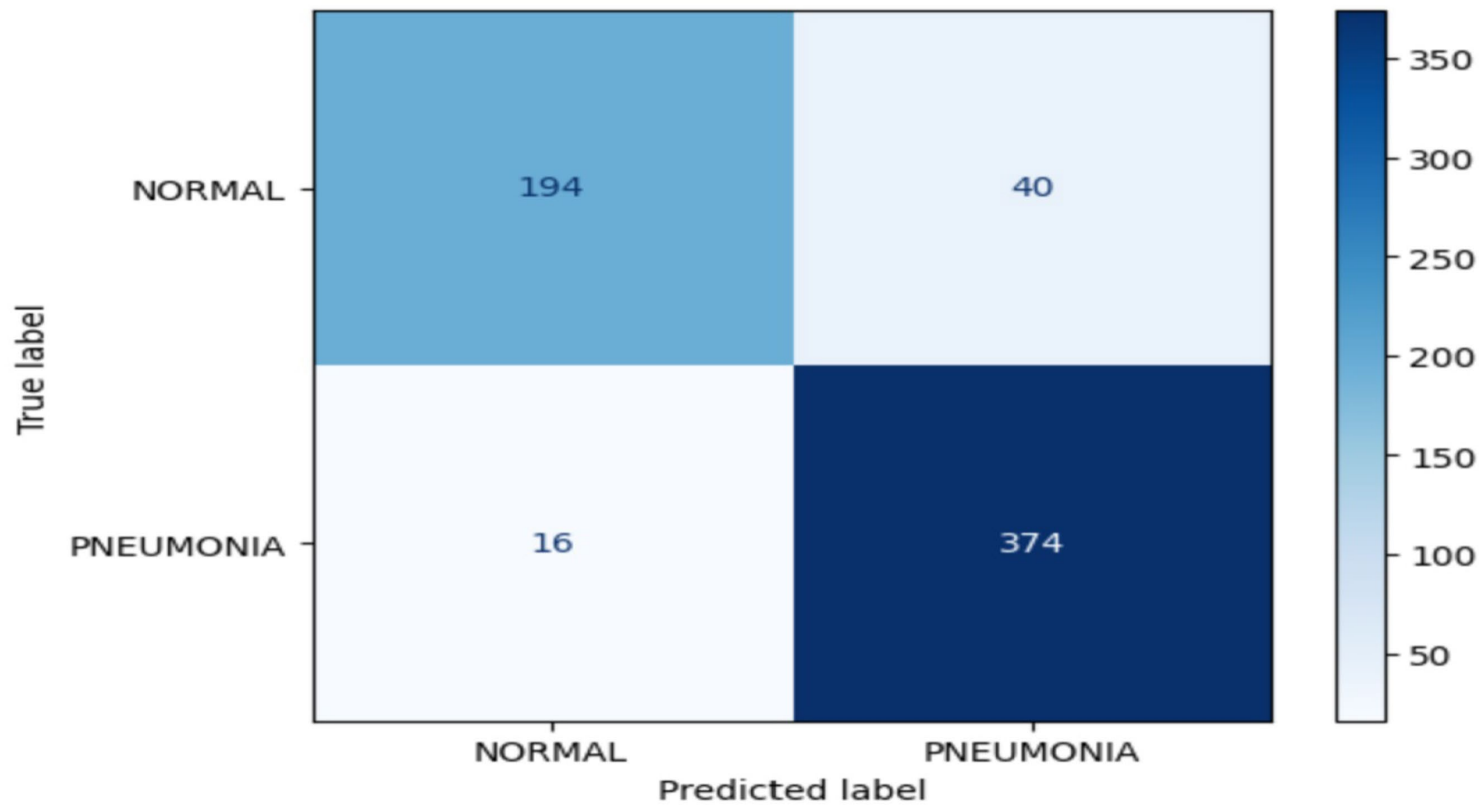
Confusion matrix for the RestNet50v2 model.

The confusion matrix for RestNet50v2 during the validation process is presented in Figure 11. This matrix is used to diagnose chest diseases. The RestNet50v2 model correctly classified194 of 234 images in the normal class, whereas 40 images were classified as false negatives in this class. Of the 390 images, 374 were classified as indicating pneumonia. Sixteen images were FP.

**Figure 12:**
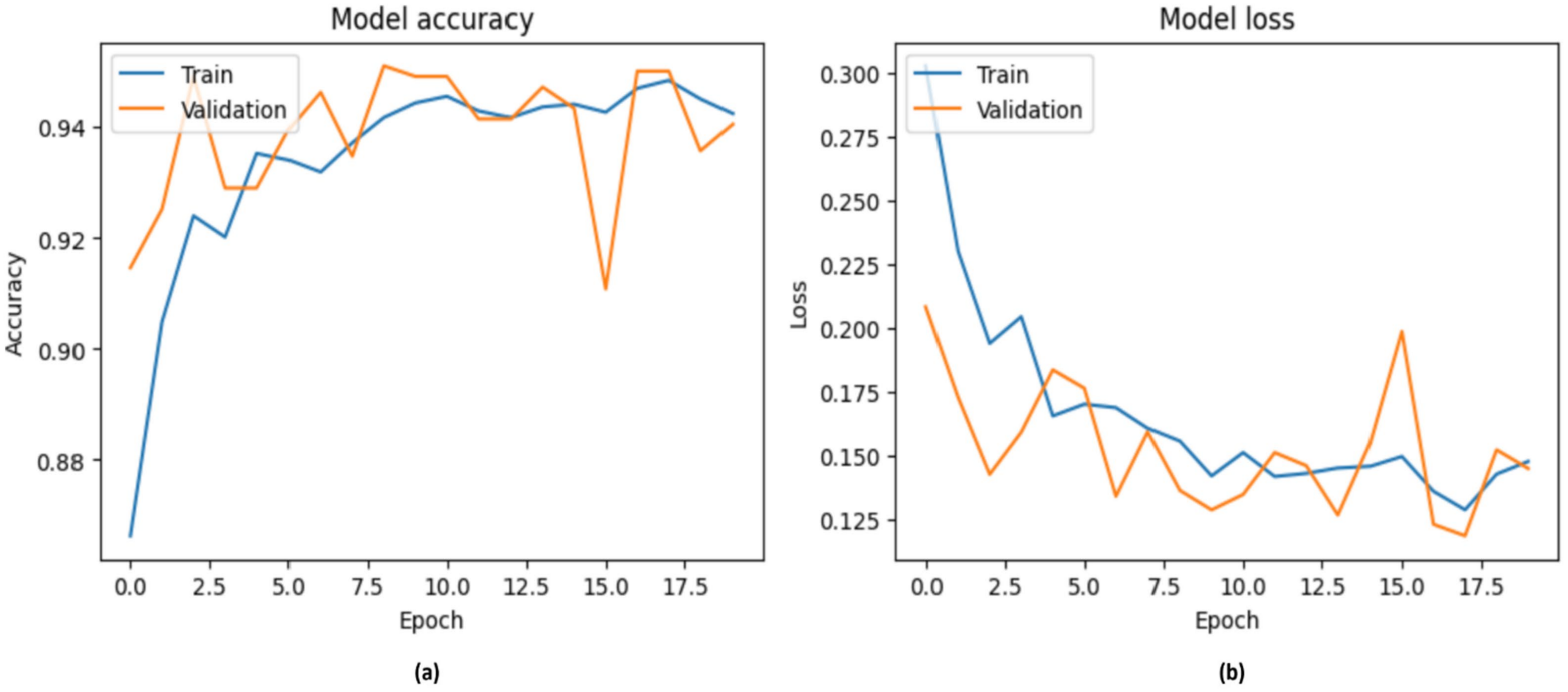
Accuracy of RestNet50v2, with **(A)** indicating accuracy and **(B)** indicating loss.

Figure 12 illustrates the performance of the RestNet50v2 model. It is noteworthy that the model attained a score of 95% in the training phase; however, during the validation phase, its performance declined from 86 to 9%. This led to a decrease from 0.3000 to 0.125 in the model’s loss rate during the validation phase.

## 5 Discussion

According to the World Health Organization, more than 4 billion individuals do not have access to expert knowledge about medical imaging, highlighting the potential value of such resources. An automated system for interpreting chest X-rays could be particularly beneficial, even in well-developed healthcare systems. This approach can help prioritize tasks, facilitating swift diagnosis and treatment for critically ill patients, especially in hospitals where radiologists are not immediately accessible. Moreover, even seasoned radiologists are vulnerable to human limitations, such as emotional fatigue and cognitive biases, which can result in diagnostic errors.

The study results indicate that DL can enhance algorithms for autonomously recognizing and localizing various conditions in chest X-rays at a proficiency level comparable to that of experienced radiologists. The clinical integration of this technology might transform patient care by reducing diagnostic times and making chest X-ray interpretations more accessible to patients. In this experiment, a chest X-ray dataset was obtained via a Kaggle competition including public participants. Image data generators facilitated the training, validation, and testing of a DL using chest X-ray images, which were resized to 224 × 224 pixels. The use of random changes in the training data facilitated improved model generalizability and mitigated overfitting. These transformations include rotation, translation, scaling, and reflection. The validation split option allocates 20% of the training data for validation purposes. To achieve normalization, only the pixel values are rescaled, and the test data are imported separately, without any augmentation. This arrangement facilitates the training, validation, and testing of a binary classification model. During the resizing process, the pixel values of the images are normalized to a range of 0 to 1. This normalization is crucial in standardizing the input data, allowing DL models to interpret the images effectively. The transfer learning architectures MobileNetV2, VGG-16, and ResNet50V2 were used to address the image features obtained from the preprocessing stages for classification purposes.

In conclusion, the MobileNetV2 model achieved 92% accuracy, surpassing the VGG-16 and ResNet50V2 methodologies in this regard. The RestNet50V2 model attained a score of 91% across several accuracy metrics. The proposed framework is assessed against many contemporary systems using identical datasets. Consequently, the system attained a significant level of accuracy. Table 7 provides a comparative analysis of the system’s results, along with several chest diagnostic methods developed by researchers over the years.

**Table 7:** Results of DL models beside existing chest diagnosis systems.

Reference	Dataset	No. of images	Models	ACC
Varshni et al. (33)		Same dataset, 1,431 images	AlexNet, GoogLeNet, and ResNet	90%
Szepesi and Szilágyi (34)		Same dataset, 5,856 images	CNN	91%
Raje and Jadhav (35)		Same dataset, 2,756 images	ResNet50v2	87%
Proposed		Same dataset, 5,856 images	MobileNet V2	92%

**Figure 13:**
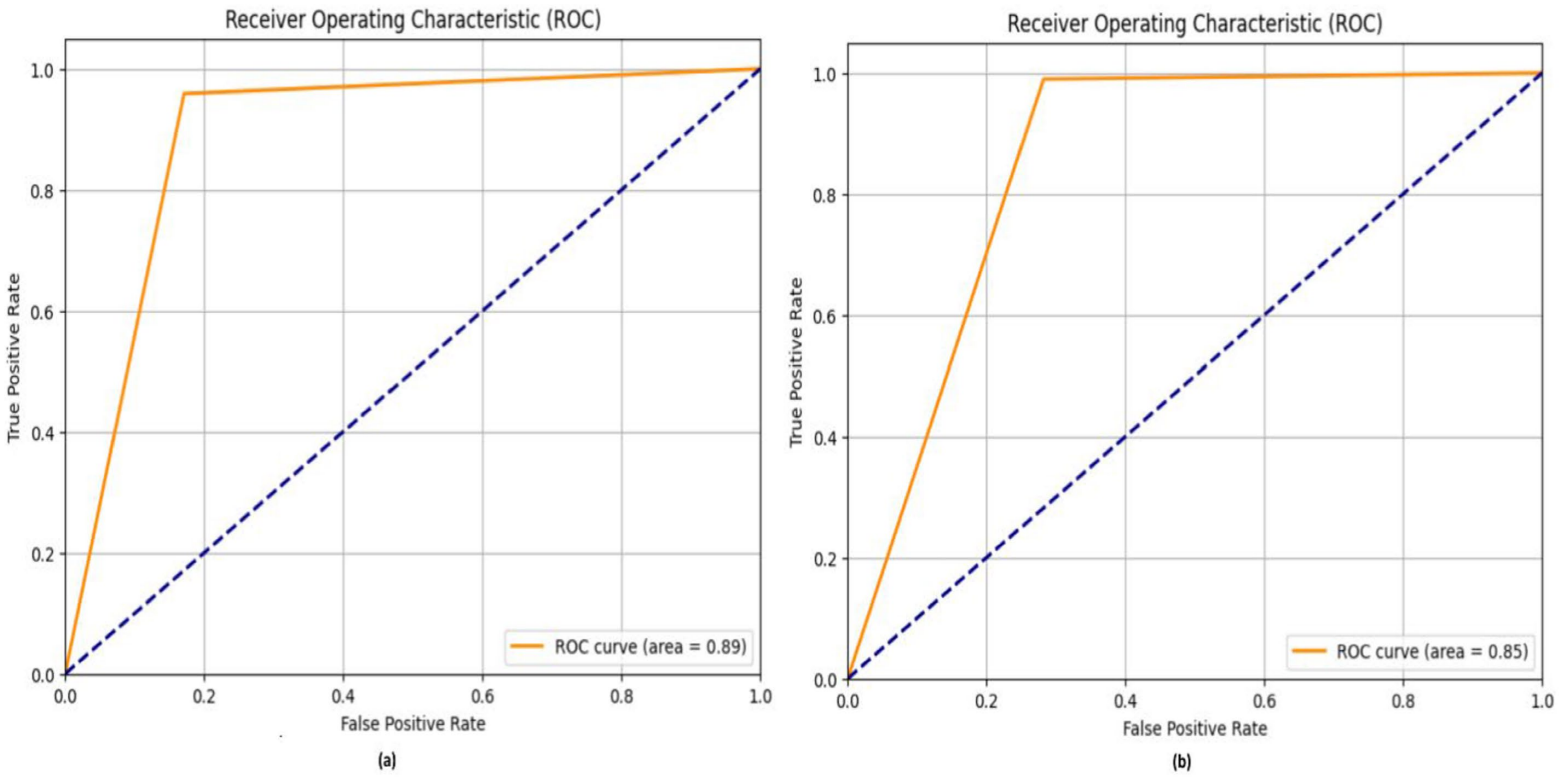
ROC of DL models **(a)** MobileNetV2 and **(b)** ResNet50V2.

The TP rate is often shown on the *y*-axis of ROC curves, while the false positive rate is depicted on the *x*-axis. This indicates that the optimal spot has been attained. Although this may not be especially pragmatic, it suggests that a large area under the curve (AUC) is often advantageous. Figure 13 shows the ROC of the proposed DL models for diagnosis chest disease.

## 6 Conclusion

Deep learning has emerged as one of the most effective techniques for the evaluation and processing of medical images in recent years. The many available methods and solutions facilitate the creation of technologies that aid doctors in the prediction and prevention of diseases at an early stage. A very promising research area at the convergence of medicine and computer science is medical image processing using DL techniques. In this research project, we developed and tested three DL systems, namely MobileNetV2, VGG-16, and ResNet50V2, that classified clinically significant anomalies on chest radiographs with a performance level comparable to that of practicing radiologists. During prospective evaluations in clinical settings, the algorithm may allow access to chest radiograph diagnostic procedures for large numbers of patients. In this experiment, we assessed three distinct DL models using a dataset of 5,863 X-rays that were categorized into two classes: normal and pneumonia. The experiment produced encouraging results, with the MobileNetV2 model achieving 92% accuracy and ResNet50V2 demonstrating 91% accuracy. The performance of each model was commendable, indicating that the use of more sophisticated approaches and the enhancement of their learning capacities may be very advantageous. This suggested system has the capacity to enhance healthcare delivery and expand access to expertise in chest radiography, allowing the detection of various acute conditions. Additional research is needed to assess the practicality of these findings in future clinical environments. In order to make models more accurate, the transformers models will be used in future.

## Data Availability

The original contributions presented in the study are included in the article/supplementary material, further inquiries can be directed to the corresponding authors.
